# Quantitative Assessment of 2q35-rs13387042 Polymorphism and Hormone Receptor Status with Breast Cancer Risk

**DOI:** 10.1371/journal.pone.0066979

**Published:** 2013-07-22

**Authors:** Chao Gu, Liang Zhou, Jianping Yu

**Affiliations:** Department of General Surgery, Jinshan Hospital, Fudan University, Shanghai, People's Republic of China; MOE Key Laboratory of Environment and Health, School of Public Health, Tongji Medical College, Huazhong University of Science and Technology, China

## Abstract

**Background:**

The association between rs13387042 polymorphism on 2q35 and breast cancer (BC) has been widely evaluated since it was first identified through genome-wide association approach. However, the results have been inconclusive. To investigate this inconsistency, we performed a meta-analysis of all available studies dealing with the relationship between the 2q35-rs13387042 polymorphism and BC.

**Methods:**

Databases including MEDLINE, PubMed, EMBASE, ISI web of science and CNKI (China National Knowledge Infrastructure) were searched to find relevant studies. Odds ratios (ORs) with 95% confidence intervals (CIs) were used to assess the strength of association. The random-effects model was applied, addressing heterogeneity and publication bias.

**Results:**

A total of 24 articles involving 99,772 cases and 164,985 controls were included. In a combined analysis, the summary per-allele odds ratio (OR) for BC of 2q35-rs13387042 polymorphism was 1.13 (95% CI: 1.11–1.16; P<10^−5^). Significant associations were also detected under co-dominant, dominant and recessive genetic models. In the subgroup analysis by ethnicity, significantly increased risks were found in Asians, Caucasians and Hispanic whites for the polymorphism in all comparisons; whereas no significant associations were found among Africans. In addition, we find 2q35-rs13387042 polymorphism conferred significantly risks for both ER-positive and ER-negative tumors. Furthermore, significant associations were also detected both in PR–positive and PR–negative cancer.

**Conclusions:**

Our findings demonstrated that rs13387042-A allele is a risk-conferring factors for the development of BC, especially in Asians, Caucasians and Hispanic whites.

## Introduction

Breast cancer (BC), as a substantial global public health concern, is one of the most common cancers diagnosed in women and is the primary cause of death among women in both the developing and developed world [1]. It is estimated that over one million women are diagnosed with BC every year, and more than 410,000 will die from the disease [2]. During the past two decades, there are well-documented reductions in mortality from BC in many counties. However, incidence rates continue to increase and do so more rapidly in countries that historically had low rates [3]. The etiology of BC is extremely complex and, while not yet elucidated, appears to involve numerous genetic, endocrine, and external environmental factors [4].

Family history is an important risk factor for BC. The risk of developing BC for a woman with a first-degree affected relative is increased 2-fold [5]. The risk is even greater for women with multiple cases in family members. BC may be attributable to mutations in high-penetrance genes such as BRCA1, BRCA2, p53 and PTEN, as well as moderate or low penetrance genes (e.g., CHEK2, ATM, HRAS1, BRIP1, and PALB2), but these mutations account for a relatively small proportion of the heritable risk in these BC families [6], [7]. Since 2007, several genome-wide association studies of BC [5], [8]–[10], have identified a number of genetic susceptibility loci that are associated with the risk of BC. Recently, a genome-wide association (GWA) study conducted in European ancestry population by Stacey et al. identified a new genetic susceptibility locus, rs13387042, at chromosome 2q35 was associated with BC risk [11]. After that, a number of studies have investigated the association between 2q35 rs13387042 polymorphism and BC risk. However, these studies have yielded conflicting or inconclusive result. These disparate findings may be due partly to insufficient power, phenotypic heterogeneity, population stratification, small effect of the polymorphism on BC risk, and even publication biases. Therefore, we carried out a comprehensive meta-analysis on all eligible studies to estimate the overall BC risk of 2q35-rs13387042 polymorphism as well as to quantify the between-study heterogeneity and potential bias.

## Materials and Methods

We performed this analysis in accordance with the guidelines of the Preferred Reporting Items for Systematic Reviews and Meta-analyses (PRISMA) statement [12] (Checklist S1).

### Literature search strategy and inclusion criteria

Epidemiological genetic association studies published before the end of December 2012 on breast cancer and polymorphism in the chromosome 2q35 were sought by computer-based searches from databases including MEDLINE, PubMed, EMBASE, ISI web of science and CNKI (China National Knowledge Infrastructure) without language restriction. Search term combinations were keywords relating to the chromosome 2q35 (e.g., “2q35”, “rs13387042”) in combination with words related to breast cancer (e.g., breast cancer' or ‘malignant breast neoplasm’). All searched studies were retrieved, and their bibliographies were checked for other relevant publications. Review articles and bibliographies of other relevant studies identified were hand-searched to find additional eligible studies.

Articles were included in this meta-analysis if they (a) examined the hypothesis that 2q35-rs13387042 polymorphism was associated with BC risk, (b) followed a case-control or cohort study design, (c) identify BC cases histologically or pathologically, and (d) provided sufficient information on genotype/allele counts between cases and controls to estimate the odds ratio (OR) and the corresponding 95% confidence interval (95% CI). The major reasons for exclusion of studies were (a) overlapping data, (b) case-only studies, (c) familiar based studies and review articles.

### Data extraction

Information was carefully extracted from all eligible publications independently by two of the authors according to the inclusion criteria listed above. The following variables were extracted from each study if available: the first author, published year, study design, geographic area, ethnicity, mean age of cases and controls, case-control match status, definition and numbers of cases and controls, source of controls, genotyping method, frequency of genotypes, and Hardy–Weinberg equilibrium (HWE) in controls. Relevant clinical characteristics included estrogen receptor (ER) status, progesterone receptor (PR) status, ERBB2 status, and tumor grade. Review reports from the two were than compared to identify any inconsistency, and differences were resolved by further discussion among all authors. Studies with different ethnic groups were considered as individual studies for our analyses.

### Quality assessment: extended-quality score

For association studies with inconsistent results on the same polymorphisms, the methodological quality should be assessed by appropriate criteria to limit the risk of introducing bias into meta-analyses or systematic reviews. A procedure known as ‘extended-quality score’, has been developed to assess the quality of association studies. The procedure scores each paper categorizing it as having ‘high’, ‘median’ or ‘poor’ quality. Detailed procedure of the quality assessment was previously described [13].

### Statistical methods

Deviation from Hardy–Weinberg equilibrium for controls was examined by χ2 tests with 1 degree of freedom. OR with 95% CIs was used to assess the strength of association between the 2q35-rs13387042 polymorphism and BC risk. The meta-analysis examined the association between the polymorphism and the risk of BC for the: (1) allele contrast, (2) heterozygous, (3) homozygote, (5) dominant, and (6) recessive model. Heterogeneity across individual studies was calculated using the Cochran's Q-statistic test followed by subsidiary analysis or by random-effects regression models with restricted maximum likelihood estimation [14]–[16]. The Q test was also performed to detect heterogeneity between subgroups. Random-effects and fixed-effect summary measures were calculated as inverse-variance–weighted average of the log odds ratio. The results of random-effects summary were reported in the text because it takes into account the variation between studies. Sources of heterogeneity were investigated by stratified meta-analyses based on ethnicity, sample size (No. cases ≥1000 or, <1000), ER and PR status. In addition, ethnicity, sample size, genotyping method and quality score were analyzed as covariates in meta-regression. The significance of the overall OR was determined by the Z-test. Publication bias was assessed with the Begg test [17] and Egger test [18]. Sensitivity analysis was performed by removing each individual study in turn from the total and re-analyzing the remainder. This procedure was used to ensure that no individual study was entirely responsible for the combined results. Statistical power (nominal α = 0.05) of this meta-analysis based on overall sample size was calculated with the pooled OR estimate from different ethnicity and minor allele frequency in controls [19]. The analyses were carried out by using the STATA software version 10.0 (Stata Corporation, College Station, TX). The type I error rate was set at 0.05. All P-values were two-tailed.

## Results

### Characteristics of included studies

Study selection process was shown in Figure S1. A total of 24 studies with 99,772 cancer cases and 164,985 controls were retrieved based on the search criteria for BC susceptibility related to the 2q35-rs13387042 polymorphism [11], [20]–[42]. In addition, all studies indicated that the frequency distributions of genotypes in the controls were consistent with Hardy–Weinberg equilibrium. The extended-quality scores ranged from 5 to 8, and 4 studies were given median quality, whereas 20 were given high quality. No ‘poor quality’ study was found. The statistical power of this meta-analysis based on overall sample size was 93%. The main study characteristics were summarized in Table 1.

**Table 1:** Characteristics of studies included in a meta-analysis of the association between 2q35-rs13387042 and BC.

Reference	Year	Country	Ethnicity	Cases/controls	Matching criteria	Genotyping method	Quality score
Dai [20]	2012	China	Asian	1771/1851	Age and region	TaqMan	High
Lin [21]	2012	China	Asian	88/69	Age	SNP Array	Median
Sueta [22]	2012	Japan	Asian	697/1394	Menopausal status and age	TaqMan	Median
Kim [23]	2012	Korea	Asian	2257/2052	Age and region	SNP Array, TaqMan	High
He [24]	2012	Europe, USA	Caucasian	3683/34174	Ethnicity and age	TaqMan	High
Harlid [25]	2012	Sweden, Iceland, Poland	Caucasian	3393/4837	Age	MassARRAY	High
Huo [26]	2012	Nigeria	African	1509/1383	Age	GoldenGate	High
Shan [27]	2012	Tunisia	African	640/367	Age	TaqMan	Median
Fletcher [28]	2011	UK	Caucasian	7643/7443	Ethnicity, age and postmenopausal hormone use	SNP Array, GoldenGate	High
Stevens [29]	2011	Europe, Australia, USA	Caucasian	2977/4976	Ethnicity and age	iPLEX	High
Teraoka [30]	2011	Denmark, USA	Caucasian	704/1386	Ethnicity, age and region	Golden Gate	High
Li [31]	2011	Sweden, Finland	Caucasian	1557/4584	Ethnicity, age and region	SNP Array	High
Jiang [32]	2011	China	Asian	492/510	Ethnicity and age	SNaPshot	Median
Chen [33]	2011	USA	African	3016/2745	Ethnicity and age	SNP Array	High
Hutter [34]	2011	USA	African	316/7484	NA	SNP Array	High
Slattery [35]	2011	USA	Caucasian, Hispanic white	1733/2041	Ethnicity and age	TaqMan	High
Campa [36]	2011	USA, Europe	Caucasian, Hispanic white, Asian, African	8314/11589	Ethnicity and age	Taqman	High
Reeves [37]	2010	UK	Caucasian	10306/10393	Ethnicity, age and region	TaqMan	High
Zheng [38]	2010	Chinese	Chinese	3039/3082	Age	SNP Array	High
Barnholtz-Sloan [39]	2010	USA	African	1230/1117	Ethnicity and age	GoldenGate	High
Antoniou [40]	2009	Europe, Australia, USA, Canada	Caucasian	7805/6675	Ethnicity, age and region	TaqMan, iPLEX, Sequencing	High
Milne [41]	2009	Europe, Australia, USA, China, Korea	Caucasian, Asian	31511/35969	Ethnicity, age and region	iPLEX	High
Zheng [42]	2009	USA	African	810/1784	Age	Massarray	High
Stacey [11]	2008	Iceland, Sweden, Holland, Spain	Caucasian	4420/17365	Ethnicity and age	Microarray, Nanongen Centaurus assays	High

NA: not applicable.

### Quantitative synthesis

**Figure 1:**
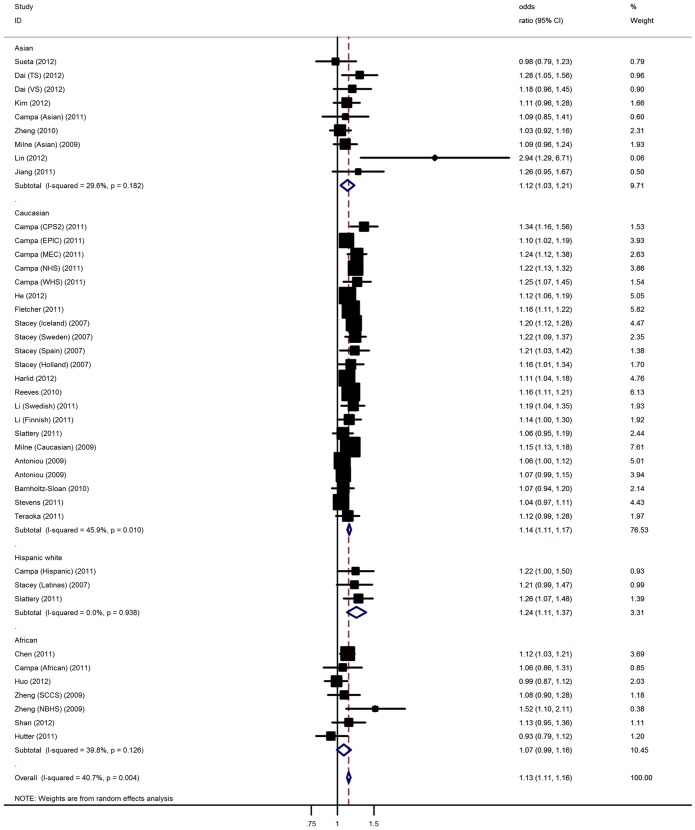
Forest plot for association of 2q35-rs13387042 polymorphism and BC risk.

Table 2 listed the main results of this meta-analysis. Using random effect model, the per-allele overall OR of the A variant for BC was 1.13 (95% CI: 1.11–1.16, P<10^−5^; Figure 1], with corresponding results for heterozygous and homozygote of 1.13 (95% CI: 1.10–1.15, P<10^−5^) and 1.20 (95% CI: 1.16–1.25, P<10^−5^), respectively. Significant associations were also found under dominant [OR = 1.12, 95% CI: 1.10–1.15, P<10^−5^] and recessive [OR = 1.19, 95% CI: 1.14–1.26, P<10^−5^] genetic models (Table S1).

**Table 2:** Results of meta-analysis for 2q35-rs13387042 polymorphism and BC risk.

Overall and subgroups analyses	No. of cases/controls	A vs. G				AG vs. GG				AA vs. GG			
		OR (95%CI)	P	P(Q)^a^	P(Q)^b^	OR (95%CI)	P	P(Q)^a^	P(Q)^b^	OR (95%CI)	P	P(Q)^a^	P(Q)^b^
Overall	99772/164985	1.13 (1.11–1.16)	<10^−5^	0.004		1.13 (1.10–1.15)	<10^−5^	0.15		1.20 (1.16–1.25)	<10^−5^	<10^−5^	
Ethnicity					0.006				0.01				<10^−4^
Asian	11681/11773	1.12 (1.03–1.21)	0.004	0.18		1.11 (1.03–1.20)	0.006	0.30		1.17 (1.04–1.32)	0.008	0.68	
Caucasian	80040/137476	1.14 (1.11–1.17)	<10^−5^	0.01		1.13 (1.11–1.16)	<10^−5^	0.15		1.21 (1.16–1.26)	<10^−5^	<10^−5^	
African	6692/14193	1.07 (0.99–1.16)	0.07	0.13		1.06 (0.97–1.16)	0.19	0.34		1.10 (0.93–1.29)	0.27	0.10	
Hispanic white	1359/1543	1.24 (1.11–1.37)	<10^−4^	0.94		1.25 (1.09–1.44)	0.001	0.63		1.31(1.12–1.53)	0.001	0.45	
Sample size					0.24				0.13				0.09
<1000	12459/27506	1.18 (1.13–1.23)	<10^−5^	0.22		1.16 (1.11–1.21)	<10^−5^	0.45		1.22 (1.14–1.30)	<10^−5^	0.12	
≥1000	87313/137479	1.12 (1.09–1.15)	<10^−5^	0.003		1.12 (1.09–1.16)	<10^−5^	0.09		1.20 (1.14–1.26)	<10^−5^	<10^−5^	

a Q statistic test used to assess the heterogeneity in subgroups.

b Q statistic test used to assess the heterogeneity between subgroups.

Significant heterogeneity was present among the included studies of the rs13387042 polymorphism (P<0.05). Ethnicity (P = 0.002) and sample size (P = 0.03) explained a large part of the heterogeneity, whereas genotyping method (P = 0.47), genotyping method (P = 0.23), and quality score (P = 0.55) explained little heterogeneity. In view of significant heterogeneity and to seek for its potential sources, we performed a panel of subgroup analyses on ethnicity and sample size. When stratifying for ethnicity, an OR of 1.12 (95% CI: 1.03–1.21, P<10^−5^) and 1.14 (95% CI: 1.11–1.17, P<10^−5^) resulted for rs13387042-A variant, among Asians and Caucasians, respectively. Significant associations were also found among Hispanic white with a per-allele OR of 1.24 (95% CI: 1.11–1.37, P<10^−4^), while no significant associations were detected in African populations for the polymorphism. Since, between-study heterogeneity decrease significantly, ethnicity was identified as a main source of heterogeneity. By considering sample size subgroups, the OR was 1.18 (95% CI: 1.13–1.23, P<10^−5^) in small studies compared to 1.12 (95% CI: 1.09–1.15, P<10^−5^) in larger studies. Similar results were also detected under co-dominant, dominant and recessive genetic models.

### Interactions between rs13387042 and hormone receptor status with BC risk

Because ER and PR status is one of the major markers of BC subtypes, we further performed analyses to test for differences in the associations of the polymorphism with BC risk with respect to different ER and PR status (Table 3). Stratification of tumors by ER status indicated that rs13387042 polymorphism increased risk of both ER-positive and ER-negative tumors. However, stronger association was observed with ER-positive tumors 1.17 (95% CI: 1.15–1.19, P<10^−5^) compared to ER-negative tumors 1.08 (95% CI: 1.04–1.13, P<10^−4^). In addition, 2q35-rs13387042 was associated with greater risk of PR-positive BC (OR = 1.18, 95% CI: 1.15–1.21, P<10^−5^) than PR-negative BC (OR = 1.10, 95% CI: 1.05–1.15, P<10^−4^).

**Table 3:** Per-allele OR for rs13387042-A variant and BC risk stratified by hormone receptor status.

Hormone receptor	Overall and subgroup analysis	No. of cases/controls	OR (95%CI)	P	P(Q)^a^	P(Q)^b^
ER	Positive	32599/96090	1.17 (1.15–1.19)	<10^−5^	0.39	<10^−4^
	Caucasian only	28453/86793	1.18 (1.14–1.21)	<10^−5^	0.15	
	Asian only	3239/7435	1.14 (1.04–1.24)	0.005	0.63	
	Negative	14519/98157	1.08 (1.04–1.13)	<10^−4^	0.15	
	Caucasian only	10696/88120	1.08 (1.05–1.12)	<10^−5^	0.37	
	Asian only	1828/6925	1.11 (0.90–1.37)	0.31	0.02	
PR	Positive	19194/56188	1.18 (1.15–1.21)	<10^−5^	0.57	<10^−4^
	Caucasian only	16611/51392	1.19 (1.14–1.24)	<10^−5^	0.20	
	Asian only	2416/4429	1.22 (1.08–1.37)	0.001	0.88	
	Negative	13080/58730	1.10 (1.05–1.15)	<10^−4^	0.16	
	Caucasian only	8337/49468	1.09 (1.04–1.13)	<10^−4^	0.20	
	Asian only	1537/3919	1.20 (0.95–1.51)	0.13	0.08	

a Q statistic test used to assess the heterogeneity in subgroups.

b Q statistic test used to assess the heterogeneity between subgroups.

### Sensitivity analyses and publication bias

Sensitivity analysis was performed by excluding one study at a time. The results confirmed the significant association between the rs13387042 polymorphism and the risk of BC, with ORs and 95% CIs ranging from 1.13 (95% CI: 1.11–1.15) to 1.14 (95% CI: 1.11–1.16). Begg's funnel plot and Egger's test were performed to evaluate the publication bias of literatures. As shown in Figures S2, the shape of the funnel plots seemed symmetrical, suggesting no publication bias among the studies included. The statistical results still did not show publication bias (Begg test, P = 0.24; Egger test, P = 0.77; Figure S3)

## Discussion

The pathogenesis of the development and progression of BC is far from being clear at present. Accumulated evidence suggests that it is a complex polygenic disorder for which genetic factors play an important role in disease etiology [4]. Common variation rs13387042 at 2q35 was originally identified in large GWA study in European population [11]. Since then, extensive case-control studies in different populations reported that the rs13387042 polymorphism in 2q35 has been implicated in BC risk. However, results of genetic association studies were confusing because of the difficulty in replicating significant associations. Different characteristics among studies such as ethnicities, BC subtype, definition of case and control, introduced heterogeneity and made the results of association studies hard to be interpreted. A meta-analysis aiming at finding out the origin of heterogeneity and assessing overall effects of these variants on BC was performed. This is the first comprehensive meta-analysis that examined the rs13387042 polymorphisms in 2q35 and the relationship with susceptibility for BC. Its strength was based on the accumulation of published data giving greater information to detect significant differences. In total, the meta-analysis involved 24 studies for BC that provided 99,772 cases and 164,985 controls. Overall, a significant association existed between the 2q35 rs13387042 variant and BC risk.

In the subgroup analysis, study conducted in Caucasian populations was responsible for heterogeneity, and the ORs between different genetic models and sample size were consistent. The rs13387042 showed positive association with BC in Asian, Caucasian and Hispanic white populations. However, no associations were found in African descent population. In fact, the distribution of the less common G allele varies extensively between different races, with a prevalence of ∼88% among Asians, ∼49% among Caucasians and ∼28% among African population [34]–[36]. Thus, failing to identify any significant association in Caucasians and other populations could be due to substantially lower statistical power caused by the relatively lower prevalence of G allele of rs13387042. Such result could also be due to the limited number of studies among African populations, which had insufficient statistical power to detect a slight effect or different linkage disequilibrium (LD) pattern of the polymorphism among African populations. Therefore, additional studies are warranted to further validate ethnic difference in the effect of this polymorphism on BC risk. It is possible that variation at this locus has modest effects on BC, but environmental factors may predominate in the progress of BC, and mask the effects of this variation. Specific life style environmental factors, such as estrogen exposure status and smoking habit have been already well studied in recent decades [4]. The unconsidered factors mixed together may cover the role of 2q35-rs13387042 polymorphism.

Findings from previous studies suggested that several SNPs are predominantly associated with ER+ breast cancer: TNRC9-rs3803662 [11], [37], [41], 5p12-rs4415084 [9], 5p12-rs10941679 [9], FGFR2-rs2981582 [9], [43] 8q24-rs13281615 [44]. In our results, 2q35-rs13387042 was associated with both ER+ and ER− BC. Similar risks were also observed when stratified by PR status. SNP 2q35-rs13387042 showed a strongly statistically significant association with risk in ER+ and PR+ cases compared to ER− and PR− cases. Because ER and PR status are the major markers of BC subtypes, these observations suggest that inherited risk variants of these subtypes may vary. The magnitude of the observed differences is small, and by themselves these findings are unlikely to have any immediate clinical implications. However, the observed differences provide clues to the biologic mechanisms that underpin tumor heterogeneity, which may ultimately lead to improved treatment and prevention. Since rs13387042 is located in a 90-kb region of high LD without any known genes or human RNAs, indicating that further study of the biological function of this SNP is necessary.

The strengths of this study include the very large sample size, no deviation from Hardy-Weinberg equilibrium, and the high quality of the qualified studies. However, our current study should be interpreted with several technical limitations in mind. Firstly, the vast majority of white subjects in the study are of European descent, and statistical power for analyses in other ethnicities is limited. Because the sample size was considerably smaller for African studies, the main conclusions from this manuscript are based on analyses among white European and Asian women. Future studies including larger numbers of Africans are necessary to clarify the consistency of findings across ethnic groups. Secondly, our results were based on unadjusted estimates, while a more precise analysis should be conducted if individual data were available, which would allow for the adjustment by other covariates including age, menopausal status, family history, environmental factors and lifestyle [4]. Third, only published studies were included in this meta-analysis. Therefore, publication bias may have occurred, even though the use of a statistical test did not show it. Recently, two meta-analyses found that SLC4A7 and XRCC1 were associated with increased BC susceptibility [45], [46]. BC is an extremely complex disease and the same polymorphism may have different roles in different ethnicity. Using meta-analysis to combine all available evidence may help to identify new loci for BC susceptibility and thus provide insight into the in vivo relationship between candidate genes and BC. An improved understanding of the pathogenesis of BC will be beneficial in the diagnosis of prodromal symptoms and in establishing appropriate therapeutic intervention to prevent the onset and the progression of BC.

In summary, findings from this meta-analysis indicate that 2q35 rs13387042 polymorphism is significantly associated with an increased risk of BC. Further studies should investigate the markers on and adjacent to 2q35 to clarify whether the present association is causal or due to linkage disequilibrium.

## Supporting Information

Figure S1 **Flow chart of literature search for studies examining 2q35-rs13387042 polymorphism and risk of BC.**
(TIF)

Figure S2 **Begg's funnel plot of 2q35-rs13387042 polymorphism and BC risk.**
(TIF)

Figure S3 **Test publication bias of studies of the 2q35-rs13387042 polymorphism of and BC using Egger test.**
(TIF)

Table S1 **Results of meta-analysis for 2q35-rs13387042 polymorphism and BC risk under dominant and recessive genetic model.**
(DOCX)

Checklist S1 PRISMA 2009 Checklist
(DOC)
