# Supplementary figures and images for: Quantitative Assessment of 2q35-rs13387042 Polymorphism and Hormone Receptor Status with Breast Cancer Risk
Source: PLoS One. 2013 Jul 22;8(7):e66979. doi: 10.1371/journal.pone.0066979 (PMC3718795; doi:10.1371/journal.pone.0066979)

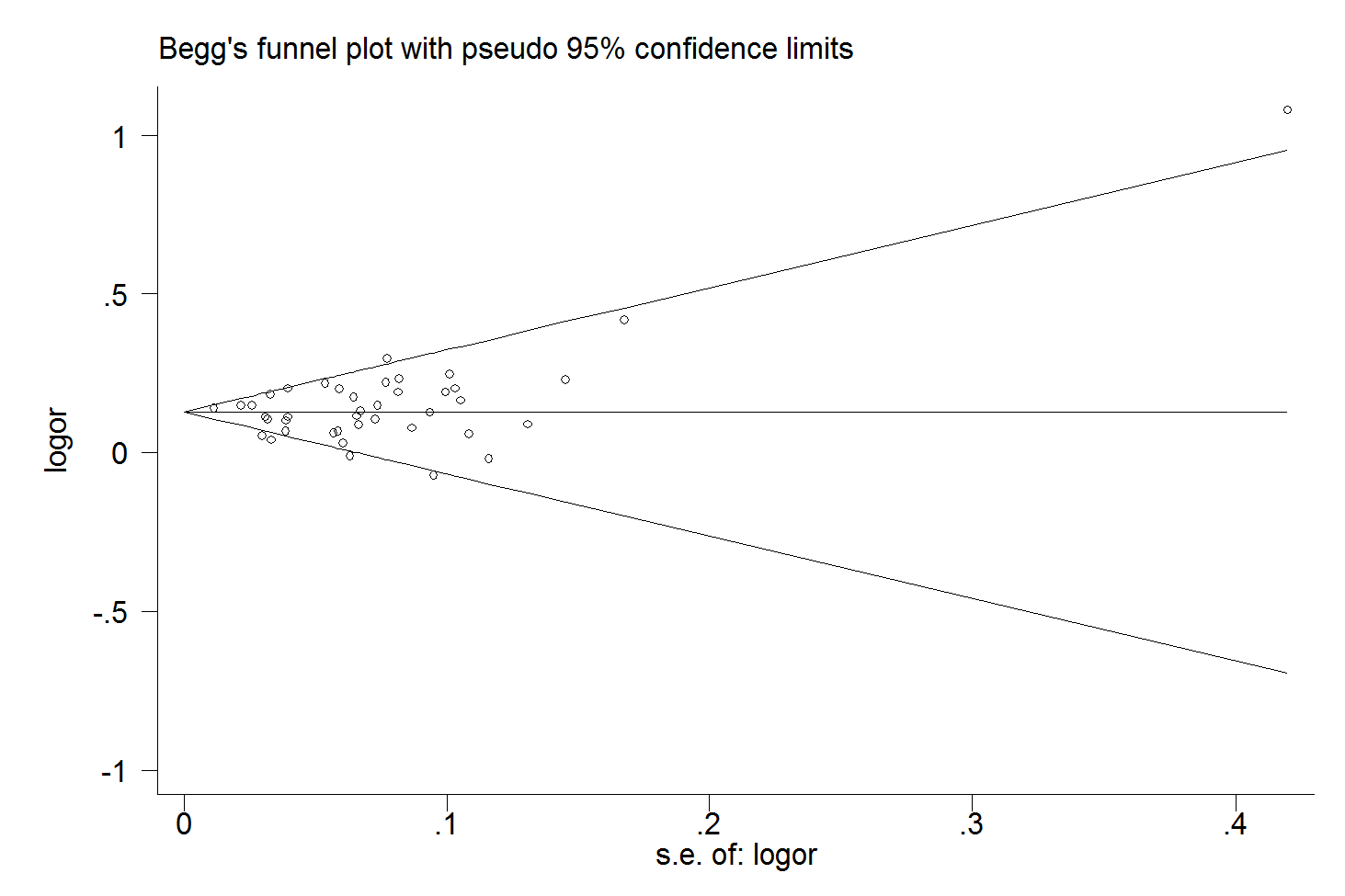

Supplement: Figure S2 — Begg's funnel plot of 2q35-rs13387042 polymorphism and BC risk. (TIF) [file pone.0066979.s002.tif]

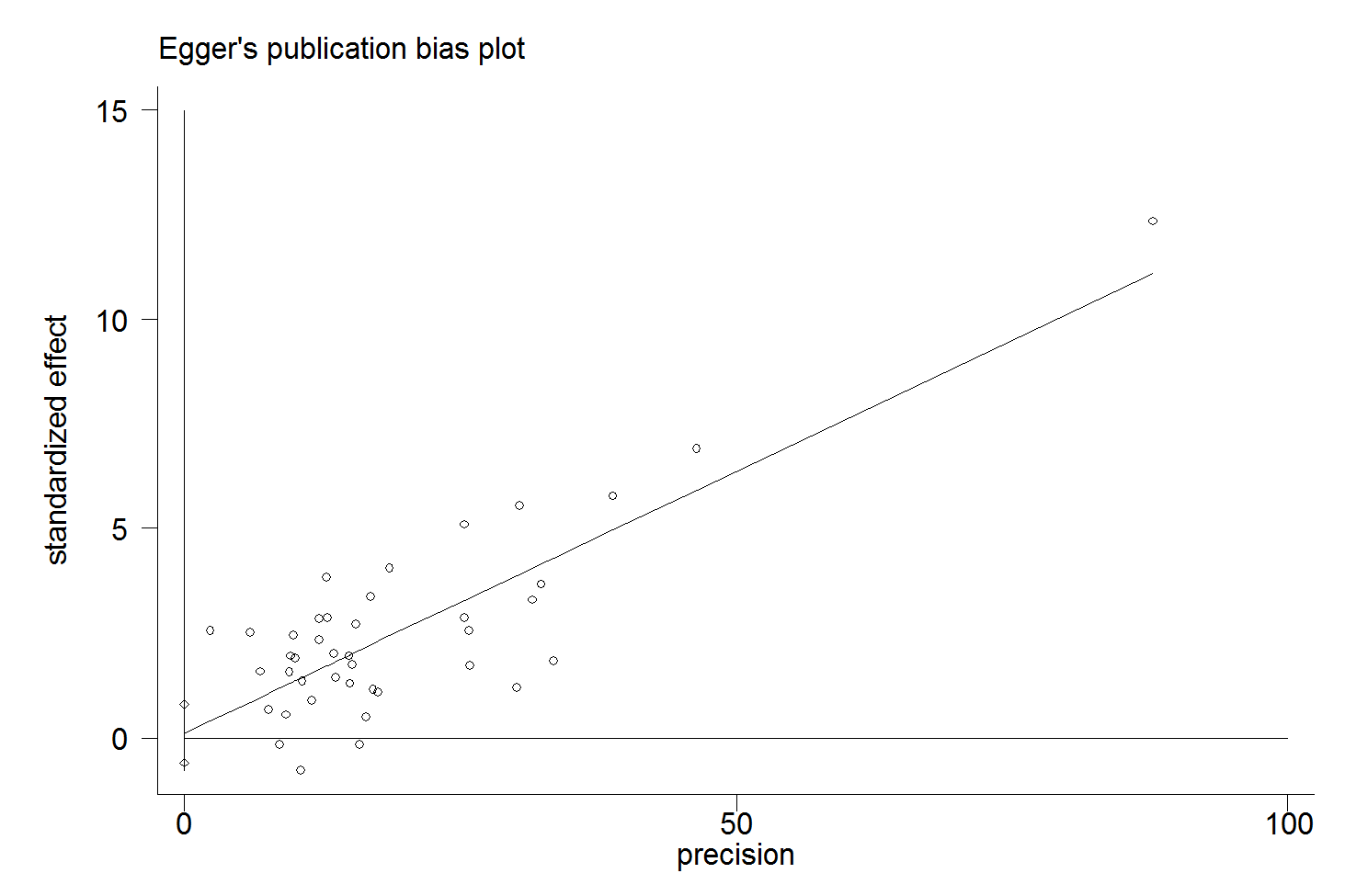

Supplement: Figure S3 — Test publication bias of studies of the 2q35-rs13387042 polymorphism of and BC using Egger test. (TIF) [file pone.0066979.s003.tif]
