# Supplementary material for: Quantitative Assessment of 2q35-rs13387042 Polymorphism and Hormone Receptor Status with Breast Cancer Risk
Source: PLoS One. 2013 Jul 22;8(7):e66979. doi: 10.1371/journal.pone.0066979 (PMC3718795; doi:10.1371/journal.pone.0066979)
Supplement: Table S1 — Results of meta-analysis for 2q35-rs13387042 polymorphism and BC risk under dominant and recessive genetic model. (DOCX) [file pone.0066979.s004.docx]

| Overall and subgroups analyses | No. of cases/controls | Dominant genetic model | | | | Recessive genetic model | | | |
| --- | --- | --- | --- | --- | --- | --- | --- | --- | --- |
|  |  | OR (95%CI) | P | P(Q)^a^ | P(Q)^b^ | OR (95%CI) | P | P(Q)^a^ | P(Q)^b^ |
| Overall | 99772/164985 | 1.12 (1.10-1.15) | <10^-5^ | 0.008 |  | 1.19 (1.14-1.26) | <10^-5^ | <10^-4^ |  |
| Ethnicity |  |  |  |  | 0.005 |  |  |  | 0.01 |
| Asian | 11681/11773 | 1.10 (1.03-1.21) | 0.003 | 0.19 |  | 1.18 (1.05-1.28) | 0.01 | 0.05 |  |
| Caucasian | 80040/137476 | 1.12 (1.10-1.16) | <10^-5^ | 0.005 |  | 1.20 (1.13-1.27) | <10^-5^ | 0.002 |  |
| African | 6692/14193 | 1.07 (0.98-1.17) | 0.10 | 0.36 |  | 1.11 (0.92-1.33) | 0.39 | 0.16 |  |
| Hispanic white | 1359/1543 | 1.23 (1.10-1.39) | <10^-4^ | 0.72 |  | 1.22 (1.06-1.51) | 0.003 | 0.28 |  |
| Sample size |  |  |  |  | 0.36 |  |  |  | 0.13 |
| <1000 | 12459/27506 | 1.14 (1.10-1.19) | <10^-5^ | 0.52 |  | 1.21 (1.18-1.31) | <10^-5^ | 0.20 |  |
| ≥1000 | 87313/137479 | 1.12 (1.09-1.16) | <10^-5^ | 0.001 |  | 1.19 (1.13-1.27) | <10^-5^ | 0.006 |  |

**Table S1** Results of meta-analysis for 2q35-rs13387042 polymorphism and BC risk under dominant and recessive genetic model.

a Q statistic test used to assess the heterogeneity in subgroups.

b Q statistic test used to assess the heterogeneity between subgroups.
